# Recent Progress in the Prevention of Serogroup B Meningococcal Disease

**DOI:** 10.1128/CVI.00566-16

**Published:** 2017-05-05

**Authors:** Ian M. Feavers, Martin C. J. Maiden

**Affiliations:** a National Institute for Biological Standards and Control, South Mimms, Potters Bar, Hertfordshire, United Kingdom; b Department of Zoology, University of Oxford, Oxford, United Kingdom; UMKC School of Medicine

**Keywords:** Neisseria meningitidis, antigenic variation, assay standardization, complement, immunity, immunization, meningitis, meningococcus, vaccines

## Abstract

The widespread use of meningococcal polysaccharide conjugate vaccines has highlighted the challenge of providing protection against serogroup B disease. Over a period of 4 decades, vaccine development has focused on subcapsular protein antigens, first with outer membrane vesicle (OMV) vaccines against epidemic outbreaks, and more recently on new multicomponent vaccines designed to offer better cross-protection against the antigenically diverse strains responsible for endemic disease. Because of the low incidence of meningococcal disease, the protective efficacy of these vaccines has not been determined in clinical studies, and their licensure has been based on serological data; however, the serological assays used to predict protective coverage have limitations. As a result, evidence of the effectiveness of these vaccines against different strains and the contribution of specific antigens to protection can only be provided by epidemiological analyses following their implementation in sufficiently large populations. The recent inclusion of the four-component meningococcal serogroup B (4CMenB) vaccine, Bexsero, in the infant immunization program in the UK has provided preliminary evidence that the vaccine is effective. Ongoing surveillance will provide valuable data on its longer-term impact and antigenic coverage. Further development of protein-based vaccines against meningococcal disease is anticipated to improve antigenic coverage and adjust to changes in circulating strains. At the same time, alternative immunization strategies may be explored to improve overall vaccine effectiveness by, for example, protecting the youngest infants or providing herd protection.

## INTRODUCTION

Meningitis and septicemia caused by serogroup B strains of Neisseria meningitidis continue to be an important health concern worldwide, despite the availability of effective vaccines against other meningococcal serogroups. In developed countries, invasive meningococcal disease occurs primarily in infants less than 1 year of age, reaching a peak at around 6 months as circulating maternal antibodies specific for the bacterium decline. The rapid onset of disease and the challenge of distinguishing it from other febrile illnesses in the very young are particular problems and make prevention through prophylactic immunization the most attractive solution. Epidemiological evidence shows that although disease most commonly occurs in infants, the meningococcus is part of the nasopharyngeal microbiome and is mainly carried asymptomatically in young adults (1). This has implications for the vaccination strategies that may be adopted to reduce infection and disease (2).

Meningococci may express one of 12 capsular polysaccharides that are defined by their immunochemistry and reflect genetic differences in their capsule loci (3). Based on their capsular polysaccharides, meningococci are assigned to serogroups, six of which are associated with invasive disease (serogroups A, B, C, W, X, and Y). The capsule is a meningococcal virulence determinant, and acapsulate meningococci do not generally cause invasive disease. Purified capsular polysaccharides were developed as licensed bivalent (A and C) and tetravalent (A, C, W, and Y) vaccines in the 1970s (reviewed in reference 4). These were, however, poorly immunogenic in infants and have subsequently been replaced by polysaccharide conjugate formulations that elicit potent antibody responses in all age groups. Other advantages of polysaccharide conjugate vaccines include their ability to disrupt transmission of the meningococcus, resulting in herd protection (5), and to overcome immunological hyporesponsiveness associated with some plain polysaccharide vaccines (6).

Although the polysaccharide conjugate vaccines arguably represent the most successful bacterial vaccine development in recent history, the development of a vaccine that will comprehensively prevent meningococcal disease has been altogether more challenging. Recently, two new vaccines have been licensed, ostensibly to provide protection against serogroup B strains. The composition of these vaccines is summarized in Table 1. Both contain the meningococcal complement factor H binding protein (FHbp), which had been identified as a candidate antigen by independent vaccine development programs at GSK in Italy (formerly Novartis) and Pfizer in the United States. The FHbp antigenic variants included in each vaccine formulation are compared in Table 2. Early in 2013, the European Commission approved the four-component meningococcal serogroup B (4CMenB) vaccine, Bexsero (GSK). This and the bivalent recombinant lipoprotein (rLP2086) vaccine, Trumenba, have subsequently been used prior to licensure in the United States, under investigational new drug applications, to respond to outbreaks of serogroup B disease among university students. Since the autumn of 2015, the 4CMenB vaccine has been part of the routine infant immunization program in the United Kingdom. Here, we review why the prevention of serogroup B disease has proven challenging, as well as the vaccine developments and issues that remain to be resolved.

**TABLE 1:** Protein-based vaccines for the prevention of group B meningococcal disease

Proprietary name	Other names or descriptions	Manufacturer	Presentation	Active ingredients (per dose)^*a*^	Amt (μg)	Adjuvant
Bexsero	4CMenB meningococcal group B vaccine (rDNA, component, adsorbed)	GSK Vaccines	Single dose, liquid suspension in a prefilled syringe	OMV from NZ98/254	25 total protein	Al(OH)_3_
				rNHBA fusion protein	50	0.5 mg of Al^3+^
				rNadA protein	50	
				rFHbp fusion protein	50	
Trumenba	rLP2086 meningococcal group B vaccine (rDNA, bivalent, lipidated component, adsorbed)	Pfizer	Single dose, liquid suspension in a prefilled syringe	Two antigenic variants of lipidated rFHbp	60 each protein	AlPO_4_, 0.25 mg of Al^3+^

a rNHBA, recombinant Neisseria heparin-binding antigen; rNadA, recombinant Neisseria adhesin A; rFHbp, recombinant factor H binding protein.

**TABLE 2:** Nomenclature of FHbp variants included in meningococcal vaccines

Vaccine	Allele (PubMLST)^*a*^	GSK variant^*b*^	Pfizer variant^*c*^	Modular groups^*d*^
Bexsero	1	1.1	B24	A1.2, B1.1, C1.5, D1.5, E1.8
Trumenba	45	3.45	A05	A1.2, B1.2, C1.1, D1.1, E1.22
	55	1.55	B01	A1.3, B1.2, C2.2, D1.1, E2.1

a https://pubmlst.org/neisseria/fHbp/ (Jolley and Maiden [74]).

b According to Masignani et al. (75).

c According to Fletcher et al. (76).

d As defined by Beernink and Granoff (77).

## IMMUNE EVASION

Since the meningococcus belongs to the normal microbiome of the human nasopharynx and is not known to have any other habitat, it is highly adapted to a commensal lifestyle and has evolved to evade host immunity. This has been a confounding factor in vaccine development. Meningococcal surface carbohydrates often mimic self-antigens, presumably allowing the organism to capitalize on host immunological tolerance to avoid immune attack. For example, the serogroup B capsular polysaccharide, an α2-8-linked polysialic acid, is similar to α2-8-sialylated human glycoproteins, such as neural cell adhesion molecules in the fetus (7). Similarly, many of the meningococcal lipopolysaccharide (LPS) structures have a terminal lacto-*N*-neotetraose structure, which is also present in paragloboside, a glycolipid found in human cells (8, 9). In addition to the various capsule and LPS structures, subcapsular outer membrane proteins and lipoproteins are often highly antigenically variable as a consequence of diversifying selection driven by host immunity. They may also be downregulated or switched off when they are not required so as not to be a target of the immune response. The meningococcal genome is replete with repeat elements, tracts of repeated nucleotides, and short nucleotide repeats, many of which are located in the coding or promoter sequences of antigen genes and regulate their phenotypic expression (10). Understanding how to overcome this genotypic and phenotypic diversity, which reflects the organism's intimate relationship with humans, has been a critical challenge for vaccine developers.

## VACCINES FOR PROTECTION AGAINST SEROGROUP B MENINGOCOCCI

Together, the perceived risk that a serogroup B polysaccharide conjugate might elicit a harmful autoimmune response and the poor immunogenicity of a candidate *N*-propionylated serogroup B polysaccharide-tetanus toxoid conjugate observed early in its clinical development (11) have effectively precluded further development of this type of vaccine. Instead, the development of vaccines to fill the gap left by the lack of a serogroup B conjugate has focused on subscapular protein antigens. The most widely used protein vaccines are based on detergent-extracted outer membrane vesicles (OMVs). Meningococcal OMVs, released naturally from the outer membrane during growth, are soluble and have the advantage of presenting outer membrane protein antigens in their native conformation (12). To date, licensed OMV vaccines have been manufactured based on a detergent extraction process, which improves yield and reduces reactogenicity by lowering the LPS content; however, the use of genetically modified meningococcal strains may in the future lead to vaccines consisting of native OMVs. Native OMVs have a potential advantage, as detergent extraction solubilizes phospholipids and membrane-associated lipoproteins that might otherwise enhance the immune response. Native OMV production has been facilitated by the development of strains with *rmpM* and *lpxL1* mutations, which improve OMV yield and reduce the toxicity of LPS, respectively (13, 14).

The clinical efficacy of OMV vaccines is primarily mediated by bactericidal antibodies to the immunodominant PorA porin (15). As a result of the antigenic diversity of PorA, this type of vaccine offers strain-specific protection; therefore it is only used to control clonal epidemics and would be of limited value for routine immunization programs. The clinical evaluation and implementation of monovalent OMV vaccines have been thoroughly reviewed elsewhere (16). In short, monovalent OMV vaccines are safe, with effectiveness estimates ranging from 54 to 83% against the homologous strain. Immunity depends on the number of vaccine doses and is age related, with cross-reactive responses to heterologous strains that are nonexistent in infants and limited in adults.

During the 1990s, candidate OMV vaccines containing multiple PorA proteins were developed to overcome the limitation of strain specificity (17, 18). Based epidemiologically on prevailing strains, six- and nine-valent formulations (Hexamen and Nonamen, respectively) have been prepared using recombinant meningococci, with each expressing three PorA subtypes. Theoretically, such vaccines offered the prospect of very high levels of coverage against the antigenically diverse strains responsible for most cases of endemic meningococcal disease (19). In clinical studies, however, some PorA subtypes appeared to be immunodominant, and these vaccines have not been developed commercially. A native OMV version of the nine-valent formulation has given encouraging results in animal studies but has not so far been tested in humans (20).

An alternative approach to overcoming the specificity of OMV vaccines has been to increase the breadth of antigenic coverage by including additional recombinant protein antigens. In the case of the 4CMenB vaccine, Bexsero, the vaccine consists of three recombinant antigens, first identified by reverse vaccinology (21), formulated with the detergent-extracted OMVs used in an outbreak-specific vaccine in New Zealand in 2004 (22). The three additional antigens are complement factor H binding protein (FHbp), Neisseria adhesin A (NadA), and Neisseria heparin-binding antigen (NHBA). The FHbp and NHBA are included as chimeric proteins fused to other meningococcal proteins. Clinical studies in infants and children demonstrated that all four components elicited specific bactericidal antibody responses to a panel of reference isolates expressing antigens that were identical to those in the vaccine (23, 24). The vaccine also proved to be safe, although it caused higher rates of fever when coadministered with routine pediatric diphtheria-tetanus-acellular pertussis–Haemophilus influenzae type b–inactivated polio vaccine (DTaP-Hib-IPV) combination vaccines.

In 2014, the 4CMenB vaccine was used to disrupt an outbreak of group B meningococcal disease among students at Princeton University in the United States. Although the outbreak strain lacked NadA and did not match the PorA subtype of the vaccine, Bexsero was expected to offer protection because of the similarity of the FHbp and NHBA components with the outbreak strain. A preliminary analysis of the impact of the vaccine, published a year after implementation, revealed that there had been no further cases among vaccinated students (25). This analysis, however, was too small to conclude whether the vaccine had provided protection against the outbreak strain. Bexsero has also been used in targeted immunization campaigns at the University of California, Santa Barbara and in the Saguenay–Lac-Saint-Jean region of Quebec (26).

Bexsero has been approved in many countries worldwide, and it has subsequently been included in various national or regional recommendations. In September 2015, the UK included the vaccine in its publicly funded national infant immunization program. As the vaccine was approved based on serological criteria rather than evidence of direct protection, this offered the first opportunity to evaluate its impact against antigenically diverse meningococci responsible for endemic disease and to identify rare adverse events in a large population (27). Ten months after the start of this immunization program, Parikh and colleagues at Public Health England analyzed the initial impact of Bexsero in vaccine-eligible infants using the screening method and showed that effectiveness prior to administration of the booster dose was 82.9% (95% confidence interval [CI], 24.1 to 95.2%) against all MenB cases (28). Compared with the average incidence rate ratio (IRR), estimated in children of the same age during the 4 years prior to the introduction of the vaccine, there was a 50% reduction in cases of MenB disease, i.e., 37 cases compared with a previous average of 74 cases (relative IRR, 0.50; 95% CI, 0.36 to 0.71) in the vaccine-eligible cohort. After adjusting for a 14% reduction in disease over the prevaccine period, this equates to a 42% reduction in cases attributable to the vaccination program itself (relative IRR, 0.58; 95% CI, 0.40 to 0.85).

A bivalent recombinant lipidated FHbp vaccine, rLP2086 (Trumenba), was approved by the FDA in October 2014 for use in those age 10 to 25 years but has yet to be approved in Europe. FHbp is expressed by most invasive meningococcal isolates as an important virulence factor, which by binding human factor H downregulates the alternative complement pathway and thus contributes to the serum resistance of the organism. Antibodies raised against this antigen therefore potentially have a dual role in complement activation, being bactericidal both directly through activation of the classical pathway and by blocking factor H binding to prevent downregulation of the alternative pathway. Although FHbp is antigenically diverse, it can broadly be divided into two genetically distinct subvariant families, and the vaccine contains a representative from each (see Table 2) (29, 30). The rationale for the development of a bivalent vaccine is that the subfamilies are immunogenically distinct, while within a subfamily, there is evidence of cross-reactivity (29). Clinical studies demonstrated that this vaccine induced bactericidal antibodies against various isolates expressing variant FHbp antigens (reviewed in reference 31). In adults, it caused some local reactogenicity and fever in a minority of subjects. In contrast, an infant study was terminated prematurely, as the vaccine caused fever in the majority of subjects (32). The effectiveness of the bivalent rLP2086 vaccine in immunization programs has yet to be assessed; however, as it is not indicated for children less than 10 years of age, the protection of infants will be dependent upon its ability to disrupt transmission in young adults and hence provide herd protection.

## ASSESSING AND PREDICTING EFFICACY

The relatively low incidence of meningococcal disease makes the design of phase three clinical protection studies impossible, and regulatory approval of meningococcal vaccines has therefore relied upon the serum bactericidal antibody (SBA) levels elicited in subjects. This long-established correlate of protection against invasive meningococcal disease is based on observations made in the 1960s by Goldschneider and colleagues, who recorded the lack of bactericidal activity in the serum of prospective cases during an outbreak of meningococcus serogroup C (MenC) disease among U.S. military recruits (33). Only 5.6% of cases had a protective SBA titer of 4 or greater to the homologous isolate, compared with 82.2% of healthy controls. As in these early studies, human serum is the appropriate complement source to use for bactericidal assays, because the meningococcus has evolved specific mechanisms of serum resistance in its human host, exemplified by the expression of a human FHbp.

In the absence of protective efficacy studies, the approval of MenC conjugate vaccines in the UK in 1999 was based on the SBA correlate of protection, adjusted for the use of baby rabbit complement in the assay, and was subsequently validated for its suitability for infant immune responses (34). In contrast with the polysaccharide-based vaccines, the application of the SBA correlate of protection to protein-based vaccines is more complex. To date, only the protective efficacy of OMV vaccines has been established; confirmation that an SBA titer correlates with protection for other protein antigens will only be possible through population-wide surveillance now that a vaccine has been implemented. Nevertheless, irrespective of whether the SBA titer provides a meaningful correlate of protection for all antigens, it is unlikely that the Bexsero and Trumenba vaccines would have received regulatory approval without the application of a threshold SBA titer as a primary endpoint in clinical studies.

The antigenic diversity of meningococcal protein antigens poses a particular problem for the evaluation of serological responses in clinical studies. The inclusion of more proteins in a vaccine formulation increases the number of bactericidal assays required to assess the breadth of antigenic coverage. In practice, the volume of serum required and the availability of a suitable human complement source limit the number of isolates that can be assessed using bactericidal assays. Vaccine manufacturers have taken a pragmatic approach to this problem by demonstrating that their vaccine elicits an SBA response to a limited number of test isolates, typically including variants homologous to the antigens in the vaccine and then assessing the breadth of coverage using antibody binding assays with larger panels of isolates.

Antibody binding is used to determine the density of an antigen on the bacterial surface, which tends to be reflected in the susceptibility of an isolate to complement-mediated killing. In the case of the Bexsero, a capture enzyme-linked immunosorbent assay (ELISA) was used to measure the amount of three vaccine components, FHbp, NHBA, and NadA, expressed by meningococcal isolates using pooled sera from vaccinees. Together with the PorA genotype, this approach has been termed the Meningococcal Antigen Typing System (MATS) and has been used to predict the potential coverage of the vaccine in different geographical regions (35, 36). In addition to evaluating the immunogenicity of the bivalent rLP2086 vaccine in SBA assays with a large panel of antigenically diverse isolates, the potential coverage of this vaccine has been estimated using flow cytometry with a monoclonal antibody that binds a conserved FHbp epitope, the so-called Meningococcal Antigen Surface Expression (MEASURE) assay (37).

Estimates of vaccine antigen coverage based on the measurement of antigen in antibody binding assays should be treated with caution. The relationship between the cell surface density of an antigen and the susceptibility of the isolate to bactericidal activity is intuitive and has been validated for a number of isolates in both assays. Even so, it is important to remember that these assays are designed to quantify the antigen expressed by the meningococcus rather than measure the antibody response of the vaccinee; therefore, they are not a substitute for the SBA titer as the correlate of protection. Apart from the cell surface density of the antigen, it is likely that other strain-to-strain differences contribute to variations in the susceptibility of the target meningococcus to complement-mediated killing. Indeed, it is notable that although MATS predicted the susceptibility of the Princeton University outbreak strain to antibodies induced by the 4CMenB vaccine, only 66% of vaccinees had protective SBA responses (38).

There are also technical limitations to the performance and standardization of both the bactericidal and antibody binding assays. The identification of reliable source of human serum as the complement used in the bactericidal killing assay poses a particular challenge, especially for standardization, interlaboratory consistency, and strain-to-strain comparisons (39). In the course of their life, an adult will usually have carried the meningococcus on multiple occasions and, as a result, adult sera frequently contain antibodies specific for particular meningococci. A number of factors, including the strains they have carried and the period of time elapsed between carriage and donation of the serum, will together determine the specificity, quantity, and avidity of the meningococcal antibodies. As a result, the usefulness of serum as a complement source varies from one individual to another. Serum from untreated agammaglobulinemic patients is potentially a good complement source for bactericidal assays but is too rarely available to offer a practical solution for large-scale vaccine evaluation. The depletion of IgG from pooled plasma donations has been explored as an alternative and may become an option for the evaluation of SBA responses during the clinical development of future vaccines (40). In the absence of an alternative, however, antibody responses to the 4CMenB and bivalent rLP2086 vaccines were evaluated using sera screened for their lack of reactivity to the isolate used in the assay. Consequently, the use of different sources of complement, both between studies and with different test isolates in the same study, is normal. Although baby rabbit complement was commonly used to assess serum bactericidal antibody responses during the clinical studies of meningococcal polysaccharide conjugate vaccines, it has not been used in the clinical evaluation of the 4CMenB and bivalent rLP2086 vaccines (39).

An often-overlooked limitation of the bioassays using bacterial cells is that the meningococci are grown under laboratory conditions that not only vary among laboratories but also bear little resemblance to the physiological conditions encountered within the human host. This problem is exacerbated by the genetic and antigenic diversity of meningococcal isolates. For example, the level of expression of the FHbp antigen, which correlates with susceptibility to complement-mediated killing, has been shown to vary among different meningococcal isolates by as much as 15-fold (41). The *fhbp* gene is situated downstream of the fructose-bisphosphate aldolase gene (*cbbA*). It is expressed in an oxygen-dependent manner by a fumarate and nitrate reductase (FNR)-regulated promoter situated in the intergenic region, but in some isolates, it is also transcribed as part of an iron-regulated bicistronic transcript along with the upstream *cbbA* gene (42, 43). Given this level of transcriptional complexity, together with the genetic diversity of the meningococcus, it is unlikely that meningococci grown in the laboratory closely reflect the expression of the antigen *in vivo*. The lack of a link between the levels of expression of an antigen *in vitro* and *in vivo* has clear implications for the interpretation of bactericidal killing and antibody binding assays as surrogates for protection and antigenic coverage. Only the widespread use of protein-based vaccines and analysis of their effectiveness will ultimately confirm the reliability of these surrogates.

## VACCINE ANTIGEN COVERAGE, ANTIBODY PERSISTENCE, AND AGE

Notwithstanding the various challenges associated with the SBA bioassay, it has played an important part in the clinical assessment of Bexsero and Trumenba, and the resulting immunogenicity data used for licensure have been reviewed extensively elsewhere (26, 44, 45). For each of the vaccines, the assay was applied to distinct panels of reference meningococcal isolates, which reflect their different components. In the case of Bexsero, three reference isolates were chosen so that each expressed an exact match for only one of the vaccine antigens (a reference isolate for NHBA was not used in all studies) (23, 24). For Trumenba, four reference isolates were used to represent different variants of FHbp subfamilies A and B (29).

Studies in adolescents and adults demonstrated that Bexsero was highly immunogenic after two doses. The proportion of individuals with protective titers of ≥4 against the reference isolates ranged from 98% to 100%, falling to between 77% and 94% over the subsequent 2 years (46, 47). In a recent study, individual serum samples from 20 adults had similarly high levels of SBA activity against the reference isolates, whereas only 25 to 45% of the subjects had ≥4-fold increases in responses to 10 of a panel of 15 clinically relevant test isolates (48). This small study not only confirmed that clinical isolates tended to be more resistant to the bactericidal antibody elicited by immunization with Bexsero but that SBA activity also declined significantly within 4 to 6 months of the administration of the second dose of vaccine. Trumenba also proved to be highly immunogenic in this age group, with titers exceeding the lower limit of quantitation against its panel of reference isolates in 80% to 100% of vaccinees receiving three doses of vaccine (49–51). Data from a small study of individual sera from young adults, using an antigenically diverse panel of isolates to determine SBA activity, suggest that this vaccine has the potential to offer broad protection, but this remains to be confirmed by surveillance once the vaccine is more widely used.

Although the success of pediatric immunization for the prevention of infection in the early years of life is a testament to the capacity of adaptive immunity in infants to respond to vaccination, antibody responses in the very young differ from those of adults (reviewed in reference 52). In general, infant antibody responses are weaker and of shorter duration than those elicited in adults. This is the case for meningococcal protein vaccines, where there is also evidence that potentially protective infant bactericidal antibody responses are poorly cross-reactive between variants of the same antigen compared with the responses of immunologically mature vaccinees.

Infant bactericidal antibody responses have been shown to decline relatively rapidly following initial immunization with a three-dose course of OMV-based vaccines, and a booster at about 1 year of age is necessary to maintain a level of protection comparable to that in older children (53, 54). Arguably, the more striking age-related effect was first reported in a clinical trial designed to compare the serum bactericidal responses to OMV vaccines developed against specific strains causing outbreaks of disease in Cuba and Norway. In this study in Chile in 1994, the proportion of vaccinees with >4-fold rises in bactericidal antibody titers was the same for both infant and adult groups, providing the vaccine (i.e., homologous) meningococcus was used in the assay. However, if a heterologous strain was used, the proportion of responders was lower for infants than adults and not significantly different from those receiving the control vaccine (55). Thus, infants are less likely than adults to elicit cross-protective antibody responses to OMV antigens, an observation that has also been reported for the FHbp antigen in infant studies of Bexsero (56). The more cross-reactive antibody responses seen in adults probably reflect preexisting immunity induced by carriage of the meningococcus that is boosted by the vaccination.

More recently, using a panel of 10 isogenic meningococci expressing different subvariants of the FHbp variant 1 as bactericidal antibody assay test isolates, Brunelli et al. made a similar observation with sera from clinical studies of the 4CMenB vaccine, which contains the FHbp subvariant 1.1 (57). Comparing sera taken after a three-dose course of vaccine, the sera from adult vaccinees were bactericidal to all the isolates in the panel, whereas the sera from infants were only bactericidal against subvariants 1.1 and 1.2. Following a fourth (booster) dose, the sera from infant vaccinees were weakly bactericidal with a further five FHbp subvariants. They concluded that given the variability in FHbp sequence and its level of expression, FHbp would be unlikely to provide infants with good cross-protection against meningococcal disease unless formulated in a combined vaccine with other antigens.

The poor cross-reactive bactericidal antibody titers elicited by the FHbp subvariant 1.1 appear to be at odds with published MATS data, which predict a high level of coverage among related FHbp subvariants. However, the MATS data are based on pooled sera taken at 13 months of age, after a fourth dose of vaccine, when the immune response is different from that of young infants (35). To determine their accuracy, MATS predictions will ultimately have to be compared with evidence of protection in this age group. This should soon be possible following the implementation of 4CMenB vaccine in the UK infant immunization program where, although only three doses of vaccine are given at 2, 4, and 12 months of age, the highest incidence of disease is in infants.

## FUTURE DEVELOPMENT OF MenB VACCINES

Given the limitations of the currently licensed vaccines, in terms of both the infant immune response and the antigenic diversity of circulating group B meningococci, there remains a case for the development of alternatives offering improved coverage. The only type of vaccine with a potential coverage of 100% against group B meningococci would be based on the capsular polysaccharide itself. More than 10 years ago, Stein et al. reviewed the association of antibodies to the group B meningococcal capsule with potential immunopathological effects (58). Despite the speculation surrounding the similarity between the capsular polysaccharide and polysialic residues on human cells, they found there was no evidence of such an association and concluded that clinical trials of group B conjugates could still be considered. Notwithstanding the success of polysaccharide conjugate vaccines against other serogroups, providing both direct and indirect protection, speculation about potential autoimmunity means there continues to be little appetite for the development of a group B conjugate, and next-generation vaccine development remains firmly focused on protein antigens.

Of the protein-based meningococcal vaccine, only OMV-based vaccines have proven protective efficacy to date, and potentially the most cross-protective OMV vaccine candidate, Nonamen, is neither licensed nor commercially developed; however, developments in the last 10 years offer a new perspective on the design and formulation of OMV vaccines. Evidence that the persistence of virulent meningococcal lineages and their associated antigenic types are maintained by immune selection simplifies vaccine design and suggests that a vaccine based upon multiple major antigens could be formulated to specifically target invasive strains (59). It has been proposed that a formulation containing multiple variants of integral outer membrane proteins, such as PorA and FetA, could form the basis of a broadly protective vaccine (60), and a monovalent OMV produced from a meningococcus constitutively expressing the FetA antigen has been shown to be safe and immunogenic for both PorA and FetA in a clinical study (61).

At the same time, the development of safe and immunogenic native OMV vaccines (20) offers the prospect of extending this concept to include lipoprotein antigens, like the FHbp, which are solubilized and therefore lost from conventional detergent-extracted OMV preparations. Native OMV vaccine candidates have been prepared from meningococci genetically engineered to overexpress FHbp in an *lpxL1* mutant, which expresses the less-reactogenic penta-acylated form of LPS (62). They have been shown to be safe, immunogenic, and potentially cross-protective in animal and human studies (63, 64), although no infant studies have yet been reported.

The future development of an LPS vaccine cannot be ruled out altogether. Analysis of the bactericidal antibody specificities of human sera shows that anti-LPS antibodies make a significant contribution to the overall bactericidal activity and that subsets of these antibodies are cross-reactive, binding to several different LPS immunotypes (65). Phase one clinical studies of native OMVs and purified deacylated LPS formulated in liposomes have given promising results, demonstrating good SBA responses to LPS (64, 66).

## ALTERNATIVE IMMUNIZATION STRATEGIES

While the next-generation vaccines remain an aspiration, there is a compelling case to explore the impact of different immunization strategies with the existing 4CMenB and bivalent rLP2086 vaccines. There are two areas in particular that might be considered: (i) protection of the youngest infants through maternal immunization, and (ii) obtaining indirect protection (herd immunity) by the immunization of young adults.

Most cases of group B meningococcal disease occur in the first year of life, before children in the UK program have received a full course of vaccine, with more than 25% of cases occurring before the second dose is even administered (67). This, together with the limitations of the infant immune response and the propensity of Bexsero to cause fever when coadministered with other routine pediatric vaccines, makes maternal immunization a rational alternative to an infant program. There are precedents for maternal immunization with protein vaccines. Following a sharp increase in whooping cough in 2011 to 2012, associated with a high rate of disease in infants less than 3 months of age and a notable increase in infant deaths, a program was introduced in the UK to immunize women with a pertussis vaccine in the third trimester of pregnancy. Analysis of the impact of this approach has demonstrated that it is safe and effective in infants at least up to 3 months of age and probably depends upon both maternal antibodies and reduced maternal exposure (68–70). Similarly, maternal immunization against whooping cough is recommended in the United States.

In addition to the prevention of group B disease in the youngest infants, who are not likely to be fully protected by the current immunization strategy, there are several other arguments in favor of the immunization of prospective mothers with a MenB vaccine. First, as adults elicit more broadly cross-reactive bactericidal antibody responses to OMVs and other meningococcal antigens than infants, the passively acquired maternal antibodies are likely to afford better cross-protection against antigenically diverse strains. Second, experience with other vaccines would suggest that antibody responses are likely to be stronger and more persistent in adults than in infants. Third, vaccine-related fever is less common as an adverse event among adults than in infants. Fourth, maternal immunization would require fewer doses of vaccine, which, together with the lack of fever and prevention of more cases in the very young, could have a positive impact on the cost-effectiveness of the vaccine. On the downside, there are currently no data on the persistence of maternal antibody in the infant. Limited evidence from the use of other vaccines, as well as the incidence of meningococcal disease in infants itself, suggests that the half-life of maternal antibodies is short, and protective levels may not be maintained for long. In addition, there is evidence that antenatal immunization blunts the infant immune response to subsequent vaccination with some antigens (71, 72). There are currently no clinical data to indicate whether this would be an issue for either of the meningococcal protein vaccines, so further research would be needed if maternal immunization were to be considered.

The outstanding success of polysaccharide conjugate vaccines is largely attributed to their ability to reduce asymptomatic carriage and thereby to provide indirect protection (herd immunity). As transmission of the meningococcus occurs primarily among young adults, strategies that focus only on infant immunization will clearly fail to provide herd immunity. It is therefore rational to consider an immunization strategy based on the vaccination of teenagers and young adults, which, like maternal immunization, has the potential to circumvent the shortcomings of infant immunity. Unfortunately, there is little evidence of the impact of either 4CMenB or rLP2086 vaccine on meningococcal carriage to support this strategy at present. One study has demonstrated a modest reduction in meningococcal carriage among students (age 18 to 24 years) from 3 months after completing a two-dose course of 4CMenB vaccine (73). It is unclear whether this small reduction would be sufficient to provide a herd effect, which will only be determined if a vaccination program were to be widely implemented in this age group.

Whether aimed at generating maternal antibodies or indirect protection, strategies based on the immunization of young adults would have the practical advantage that both Bexsero and Trumenba are suitable for this older age group. Health authorities would have a choice of vaccines and therefore more resilience than currently exists for infant programs, where only 4CMenB vaccine is approved.

## CONCLUSION

Just as the first meningococcal polysaccharide conjugate vaccines were approved based on serum bactericidal antibody levels as a substitute for protection, the Bexsero and Trumenba vaccines have also been approved based on serological data alone. The rollout of these vaccines provides an opportunity to determine their effectiveness and validate the correlation between serological predictions and actual protection. However, the sequence diversity and variability of expression of protein antigens make surveillance, and in particular the definition of vaccine failures, an altogether more complex prospect. Using the new vaccines, there is scope to follow precedents set by other vaccinations and explore immunization strategies that have the potential to protect the youngest infants or disrupt transmission of the organism.
